# The Effects of Locality and Host Plant on the Body Size of *Aeolothrips intermedius* (Thysanoptera: Aeolothripidae) in the Southwest of Poland

**DOI:** 10.3390/insects10090266

**Published:** 2019-08-22

**Authors:** Iwona Gruss, Jacek Piotr Twardowski, Marcin Cierpisz

**Affiliations:** Department of Plant Protection, Wroclaw University of Environmental and Life Sciences, Plac Grunwaldzki 24 A, 50-363 Wrocław, Poland

**Keywords:** Thysanoptera, *Aeolothrips intermedius*, locality, host plant, body size, body mass

## Abstract

*Aeolothrips intermedius* is a thrips predator often found in phytocoenoses worldwide. Both the adults and larvae of this species prey on small invertebrates, including phytophagous species from Thysanoptera group. The aim of this study was to determine the morphological variability of the *A. intermedius* relative to the locality and, indirectly, to the species of host plant. Insects were collected from five localities in southwest Poland and five different host plants. For each of the sexes, six morphometric features were assessed: body length, length of antennae, wing length, head length, head width and length of pronotum. Additionally, the body mass for each individual was estimated. The findings revealed that in females, both the locality and host plant had a significant impact on almost all of these features. In males, the morphometric features under study correlated strongly with locality and only moderately with the host plant. Certain differences were observed between males and females, mainly in terms of antennae length. The results show that *A. intermedius* exhibits significant variability in this respect, which is indicative of the species’ phenotypic plasticity. The body length was the trait with the most distinct response to the locality and host plant.

## 1. Introduction

The majority of thrips feed on the fruit, flowers and leaves of various plants by sucking the juices out of them. Feeding by these insects causes characteristic changes in the appearance of the affected tissues. Some species also transmit plant diseases [1,2]. A small number of species in Thysanoptera, especially within the Aelothripidae family, are predatory, feeding on small invertebrates such as mites, other thrips and the larvae of some insects [3]. One of the most common predatory species found in Europe is *Aeolothrips intermedius* Bagnall, 1934 [4]. Both the larvae and adults of this species are predators, preying on the larvae of thrips and aphids, as well as the larvae and eggs of other small insects [5]. To date, *A. intermedius* has been found to prey on 44 species of thrips. Under laboratory conditions, predation on the following species of mites was also observed: *Tetranychus urticae*, *Panonychus ulmi* and *Cenopalpus pulcher*, as well as certain aphids: *Aphis fabae*, *Aphis craccivora, Acyrthosiphon pisum* and *Myzus persicae* [4]. *A. intermedius* is widespread throughout eastern and western Europe and can be found in a variety of habitats, including wild meadows and arable fields. The larvae of this species often make their way into flowers to hunt. Adult individuals supplement their diet with the pollen of the plants on which they live [6,7]. Both sexes are fully winged, though males tend to be smaller than females. Two dark transversal stripes can be seen on the first pair of wings in both males and females. The body is brown. The second and third segments of the antennae are lighter in colour, with the second segment slightly darker than the third. The antennae are nine-membered, with sensoria on the third and fourth segment. The fifth segment is longer than the total length of segments six to nine. The head and pronotum lack elongated bristles [8,9].

Morphological variation within a given insect species can fluctuate depending on a number of factors from which most important are temperature, humidity, food availability, population density and human activity. Features, such as the size of the body or its individual parts, may change to a certain extent [10,11]. The explanation of this phenotypic variation is derived from the genetic variation and phenotypic plasticity [12,13]. The genetic variability in insects occurs mostly in the large geographical scale. This was found for the peach fruit moth *Carposina sasakii* [14] or *Pseudatomoscelis seriatus* [15], both in China. On the other hand, the host plant plays the critical role in the genetic variation of the fly *Bactrocera tau* [16] and green citrus aphid *Aphis spiraecola* [17]. Considering phenotypic plasticity, it has been mostly found to be influenced by the nutrient source [18,19,20], such as in the wing shape of the cricket *Gryllus firmus* [19]. Considering thrips species, the phenotypic variation was found in a few phytophagous species to be due to the effect of geographical distance or host plant. For instance, the body size of *Thrips parvispinus* differed between lowlands and highlands [21]. The other example is the difference in the reproductive capacity of *Frankliniella occidentalis* (phytophagous thrips) in two different host plants [22]. The aim of the study was to determine the extent of morphological variability of *A. intermedius* and body mass in connection with locality and host plant.

## 2. Materials and Methods

### 2.1. Sites and Insect Sampling

**Figure 1:**
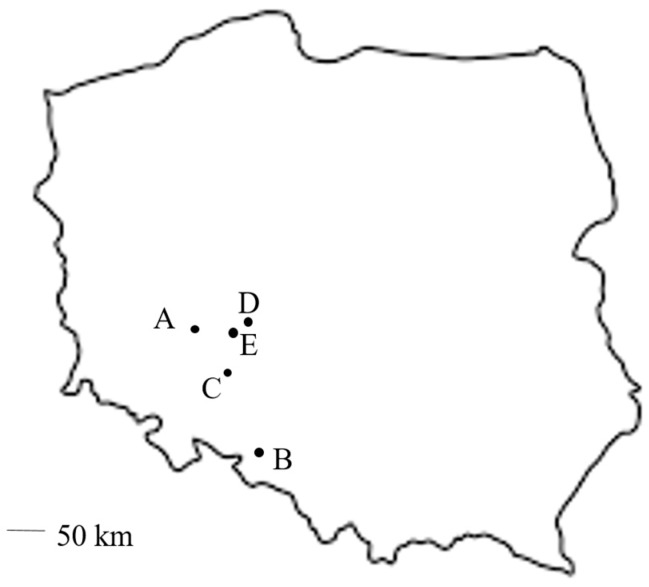
Localities of *Aeolothrips intermedius* sampling in the southwest of Poland.

Insects were sampled from five localities in Poland (Table 1). The shortest distance in a straight line between two localities was about 25 km (between localities D and E), while the greatest distances were 151 km and 156 km (between localities D and B and localities A and B, respectively) (Figure 1). Insects were collected in the first decade of July 2017 from various agricultural crops. Each of the crops were flowering during the sampling. The distance between the fields in localities 3 and 4 did not exceed 600 meters. The crops under study included three species from the Fabaceae family: soybean (different varieties), pea, narrow-leafed lupine; oilseed rape (Brassicaceae) and a mixture of flowering plants comprising 19 species, the largest share claimed by *Phacelia tanacetifolia*, *Chrysanthemum segetum*, *Trifolium pratense* and *Anethum graveolens*. The mean temperature in July varied between 16.4 °C (in locality 2) and 18.6 °C (in locality 5), and the monthly sum of precipitation was the highest in locality 1 (153 mm) and the lowest in locality 2 (95 mm). The variation between temperature and precipitation was relatively low.

In each of the localities, thrips were sampled once from a 100 m × 3 m area through the use of a sweep net. The insects were then preserved in a 75% ethanol solution. From each of the localities, 50 individuals (25 females and 25 males) of *A. intermedius* were selected for the study.

### 2.2. Morphometric Measurements

**Figure 2:**
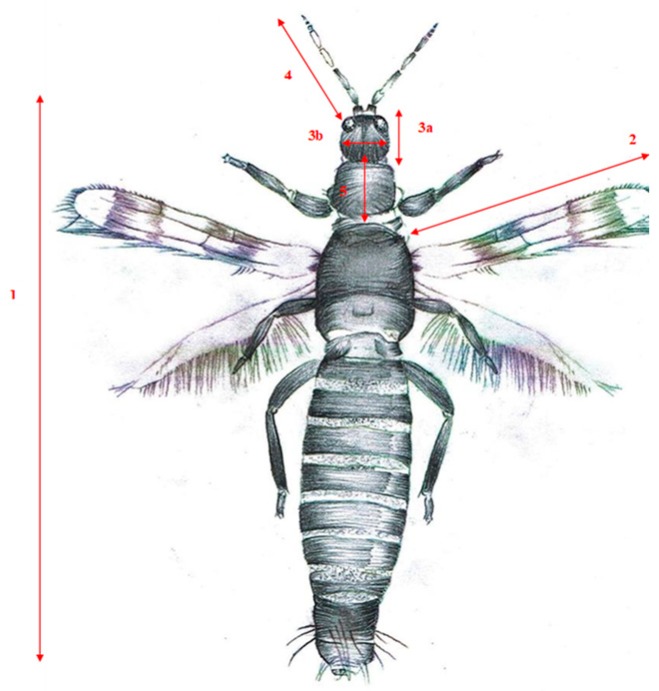
Measurements of the body parts of *Aeolothrips intermedius.* 1—body length, 2—forewing length, 3a—head length, 3b—head width, 4—antennae length, 5—pronotum length.

*Aeolothrips intermedius* was identified to the species lever under the stereoscopic microscope. In all of the samples, other thrips species were found, mainly predators. Morphometric measurements were performed on adult *A. intermedius* specimens fixed on permanent preparations using the Carl Zeiss Stemi 508 biological microscope (Carl Zeiss, Zaventem, Belgium) and the Carl Zeiss Axiocam Erc 5s camera (Carl Zeiss, Zaventem, Belgium). The ZEN 2 Core programme was used to carry out the measurements. The following features were measured: length of body from head to the end of abdomen; length of antennae from base to the end of the last segment; length of head from vertex to the tip of haustellum; head width at the level of the post ocellar setae; pronotum length and the distance between the base and the tip of the first pair of wings (Figure 2). The photographic documentation of the measurements is enclosed in Table S1. Additionally, the body mass was calculated for each specimen using the equation M (g) = aL^b1W^b2 [19], with the coefficients a = 117.7, b1 = 1.331 and b2 = 1.331, where L is the body length and W is the body width.

### 2.3. Data Analysis

The data analysis was provided separately for each sex. The question of focus was on whether the six body measurements varied with the locality and the host plant. However, particular crops (except for soybean) occurred only once in each of the localities. In order to compare the data, including all morphometric trails, the principal component analysis (PCA) was used. The analyses were conducted using PROC PRINCOMP (SAS, University Edition) (https://support.sas.com/documentation/cdl/en/statug/63347/HTML/default/viewer.htm#statug_princomp_sect004.htm). The dependent variables were 5 morphometric traits, while independent Host plant and locality. The variables were correlated with 5 principal components. The significance of the first and second principal axes in contrast to experimental treatments, was determined using linear mixed model (PROC MIXED statement) in the SAS University Edition. The effect of experimental treatments on the body mass was determined using general linear model (PROC GLM statement) (PROC GLM in SAS University Edition) (https://support.sas.com/documentation/cdl/en/statug/63033/HTML/default/viewer.htm#glm_toc.htm). In the analysis, the effects of the locality, plant and its interacting effect were included. The data, which differed significantly between each other, were compared with the Tukey’s post-hoc test.

## 3. Results

**Figure 3:**
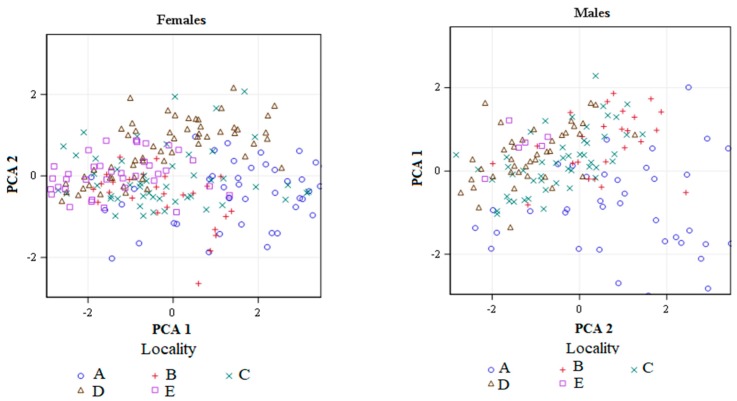

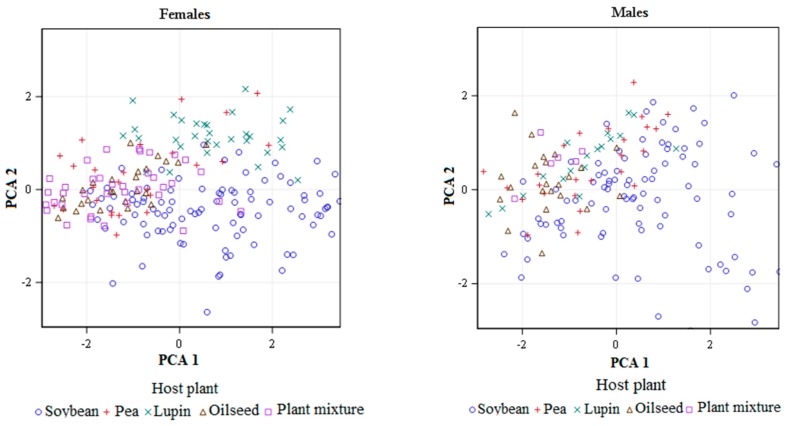
Scatter plots showing clustering of thrips in five localities and five host plants along the first and second principal component axes for males and females of *Aeolothrips intermedius.*

The morphometric traits of both sexes were sensitive to the locality and host plant (Figure 3). Two principal components explained the 78.8% variance among males and the 72.1% variance among females (Table 2). For both sexes, all morphometric features showed positive loading along the first PCA axis. The second PCA axis revealed discrepancies between head length and width (in both males and females), as well as antennae length (males only). High positive values along PCA 1 for both sexes pointed to individuals with a long head, long antennae and wings (only females), as well as a wide pronotum. PCA 2 for both sexes pointed to long-bodied specimens with a short head. Additionally, PCA 2 applied to males with short antennae and long wings. The analysis of variance showed that mean component figures for females varied with locality along PCA 1 (*p* < 0.001) and with host plant species along both axes (*p* < 0.001). The mean component figures for males varied with locality along both axes (*p* < 0.001) and with host plant species along PCA 2 (0.0215). For instance, females were clustered horizontally along PCA 1, which reflected localities, and horizontally and vertically, which reflected host plant species (Figure 3). Males were clustered horizontally and vertically reflecting locality, and vertically reflecting the host plant. In terms of locality, the morphometric traits of both sexes exhibited significant clustering around PCA 1 and PCA 2. The host plant had a significant impact on nearly all of the females’ morphometric traits clustered along the first and second PCA axis. In males, only the length of the head was clustered positively around PCA 2. Generally, considering geographic variation, the decrease in the body size was observed in the localities 3 and 5 for females, and 3, 4 and 5 for males. For both sexes, the largest individuals were found in the locality 1. Analysing the host plant effect, the smallest females were found in the plant mixture and the largest in soybean crop. Considering males, the smallest individuals were found in the plant mixture and oilseed.

**Figure 4:**
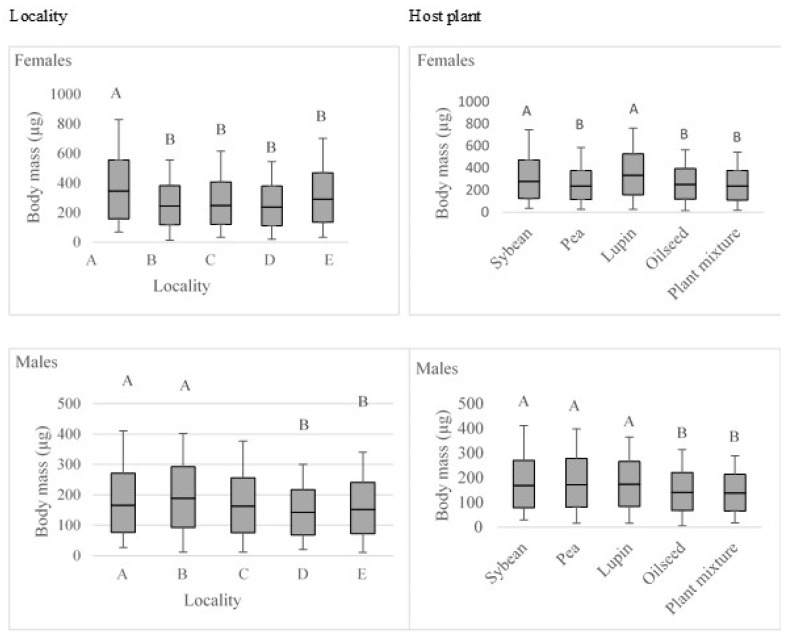
Body mass response to locality and host plant for females and males. * Different upper-case letters indicate significant differences between experimental treatments.

The body mass, which was estimated from body length and width, was significantly affected by the locality and host plant, while no interactive effects were found (Table 3). Males reached a considerably lower body mass in comparison to females (Figure 4). Female body mass was the highest in the locality 1 in comparison to the other four study sites. Males reached greater body sizes in locality 1 and 2 in comparison to localities 3, 4 and 5. Considering the host plant, the highest was found in soybean and lupin for females, and in soybean, pea and lupin for males.

## 4. Discussion

The genome and the environment are two interacting factors in forming a phenotype [23]. Thus, the adaptive changes in the morphology of an organism can be caused both by genetic variation and phenotypic plasticity [24]. Generally, body size can be an indicator of overall health in insects. Larger individuals live longer and show higher rates of reproductive success [25]. Adult size in both males and females depends on nutrition, the disruption of optimal conditions [26], dispersal ability, duration of ontogenesis and competition [27]. Some thrips species are characterized by relatively high morphological and phenotypic variability [28]. Hassall et al. [29] suggests that the wide and continuously expanding range of many thrips species is at least in part a consequence of their ability to quickly adapt to local climatic conditions. To our knowledge, this is the first attempt at characterizing predatory thrips in terms of body size in connection with locality and host plant.

The results show that both locality and host plant species had a significant impact on the body size of *A. intermedius*. Females distinctly responded to both factors, whilst males were less affected by the host plant. The distances between localities fell within the 25 and 150 km range. This was sufficient to affect the body size of *A. intermedius*. Johari et al. [21] found geographic variance in body size and colour of another thrips species—*Thrips parvispinus* in Indonesia. The morphometric variation was particularly significant between lowland and highland areas. Our research showed that all of the females’ body sizes synchronously increased or decreased depending on the locality. In males, a shorter body corresponded with an increase in the size of the antennae, wings, head and pronotum. A similar pattern was observed in the way *Phlebotomus tobbi* (Diptera) responded to temperature. In the case of this species, the wing size of females correlated negatively with temperature, while the opposite was true for males [30]. Studies on the metric features of the ground beetle *Carabus granatus* have indicated variations in body length dependent on locality in the geographic range, level of anthropogenic influence and degree of biotope openness in the Eurasia region [31]. The wing shape of some Diptera species also show geographical variation in Brazil [32]. The wing shape was found to be correlated with elevation variation, as well precipitation and temperature. A similar pattern was observed in grasshoppers living in different climates in China [33]. In this experiment, smaller individuals with shorter and blunter tip forewings were mainly distributed in lower latitudes and mountainous areas, where there are higher temperatures and more precipitation. Numerous studies have revealed that temperature is one of the most important drivers of phenotypic plasticity in insects [34,35]. The body size of *Thrips tabaci* decreases alongside increased temperature during incubation [36]. In our study, however, the mean temperature differences between the localities were quite small, indicating that the geographical variation of the morphometric features of *A. intermedius* may have resulted from conditions connected to the species’ microhabitat, including temperature and precipitation. Only in one study site—(B)—was the mean daily temperature and monthly precipitation sum considerably lower compared to other sites. The decrease in body sizes was observed in the localities C and E for females, and C, D, E for males, from which only the localities D and E were relatively close to each other. For both sexes, the largest individuals were found in the locality A.

*Aeolothrips intermedius* is mostly a predatory insect, both in the larval and adult stage. However, adults supplement their diet with the pollen of flowering field crops [7]. As such, the species’ relationship with the prey host plant is quite strong. It should be noted that the hunting grounds of *A. intermedius* are limited to the particular crop in which it lives. As a result, its body size could be related to the species, body size and availability of the prey it feeds upon [37]. Plants have different biochemical properties, some of which can render prey items nutritionally poor or even toxic, and this may affect the fecundity of their natural predators [38]. Additionally, plants exhibit a variety of ecological traits which can modify enemy–prey interactions [39]. When comparing the host plants from the Fabaceae family (soybean pea and lupin) on thrips’ body mass, similar effects were found for each of those plants in comparison to oilseed and plant mixture. In terms of the PCA analysis, the females’ response was stronger than that of the males. The effects of the host plant on the body size of herbivorous insects has long been known. For example, the species *Bactericera cockerelli* of the order Hemiptera has the capacity to significantly decrease or increase a number of its body parts [40]. In one study, the phytophagous thrips *Frankliniella occidentalis* differed in size when kept on cucumber and bean [22]. The question remains of how exactly the prey host plant influences predators on the third trophic level. In the study conducted by Giles et al. [41], the ladybird *Coccinella septempunctata* was shown to grow larger when raised on aphids kept on different prey host plants. Presumably the nutritional value of the prey influenced the adult predators’ body size [38].

## 5. Conclusions

*Aeolothrips intermedius* is a predatory thrips commonly found in a number of agricultural crops. It feeds mainly on other species from Thysanoptera. Analyses of the body size of *A. intermedius* have shown a wide range of variation. Both the locality and prey host plant significantly affected the physical characteristics of the species. However, while different localities produced distinct responses in both sexes, the host plant species appeared to mainly affect the females. Even though the distances between localities did not exceed 150 km, they were sufficient enough to cause significant variation in almost all of the measured features. In terms of host plant species, the impact was less direct and less likely to be connected with the feeding habits of the thrips prey. The high variation within the morphometric traits, particularly in body length, might be a good indicator of the changes in the agricultural ecosystems. This study provides new information on the phenotypic variation of predatory insects in relation to locality and host. Here, we confirm that adult predatory thrips change their morphometric traits as a response to the different host plants of its prey. A follow-up could be the study on the morphometric variability within larvae stages.

## Figures and Tables

**Table 1:** The characteristic of localities and host plants.

Locality	Coordinates	Crop	Varieties	Sampling Date	Mean Temperature and Monthly Precipitation Sum for July 2017
A	1. 51°.166903 N, 17°.097462 E 2. 51°.178658 N, 17°.114833 E	Soybean	Merlin Aligator	03.07.17	Temp: 17.7 °C; 153 mm
B	1. 50°.073262 N, 18°.028060 E	Soybean	Petrina	11.07.17	Temp: 16.4 °C; 95 mm
C	1. 50°.801401 N, 17°.551957 E 2. 50°.800838 N, 17°.554210 E	Soybean, Pea	Aldana James	04.07.17	Temp: 18.2 °C; 141 mm
D	1. 51°.231556 N, 17°.876641 E 2. 51°.228459 N, 17°.881705 E	Narrow-leafed lupin, Oilseed rape	Jowisz Kolumb	07.07.17	Temp: 17.9 °C; 116 mm
E	51°.144733 N, 17°.640499 E	Plant mixture	19 species of flowering plants	06.07.17	Temp: 18.6 °C; 130 mm

**Table 2:** Summary statistics from principal component analysis (PCA).

	Females				Males			
	PCA 1		PCA 2		PCA 1		PCA 2	
**Proportion of the Variance Explained**								
	68.2%		10.6%		52.47%		19.6%	
Eigenvalues								
Body length	0.37		0.67		0.22		0.71	
Head length	0.43		−0.45		0.46		−0.37	
Head width	0.41		−0.53		0.47		−0.20	
Antennae length	0.37		0.22		0.45		−0.15	
Wing length	0.42		0.12		0.30		0.54	
Pronotum length	0.43		0.07		0.48		0.04	
Significance of the axes								
	F	*p*	F	*p*	F	*p*	F	*p*
Locality	34.47	<0.001	1.47	0.2324	15.94	<0.001	26.57	<0.001
Locality × Body length	4.18	0.0271	4.40	0.0232	3.93	0.0375	1.82	0.2162
Locality × Head length	1.14	0.3210	1.56	0.0666	2.17	0.0092	2.46	0.0030
Locality × Head width	4.07	<0.001	1.93	0.0097	0.54	0.9532	2.23	0.0050
Locality × Antennae length	4.10	<0.001	1.12	03438	0.67	0.8603	1.67	0.0691
Locality × Wing length	0.69	0.7597	0.60	0.8311	1.70	0.0993	2.00	0.0469
Locality × Pronotum length	1.93	0.0137	1.86	0.0188	1.24	0.2469	4.18	<0.001
Host plant	23.24	<0.001	31.24	<0.001	2.33	0.1001	3.93	0.0215
Host plant × Body length	13.65	0.0012	7.44	0.0084	1.57	0.2838	0.15	0.9818
Host plant × Head length	2.36	0.0150	4.33	<0.001	1.76	0.0767	2.46	0.0110
Host plant × Head width	59.81	<0.001	3.11	0.0002	0.58	0.9034	0.56	0.9148
Host plant × Antennae length	4.10	<0.001	1.95	0.0430	0.48	0.9379	0.80	0.6748
Host plant × Wing length	1.30	0.2592	1.98	0.0529	0.42	0.9357	0.83	0.6160
Host plant × Pronotum length	0.57	0.8845	2.33	0.0084	1.44	0.1601	0.49	0.9309

**Table 3:** Summary table of statistics from body mass using general linear model (GLM).

	Body Mass (µg)					
Dependent Variable	Locality		Host Plant		Host Plant × Locality	
	F	*p*	F	*p*	F	*p*
Females	27.27	<0.0001	17.49	<0.0001	0.14	0.7096
Males	8.37	<0.0001	26.17	<0.0001	0.09	0.7700

## Supplementary Materials

The following are available online at https://www.mdpi.com/2075-4450/10/9/266/s1, Figure S1: Aeolothrips intermedius males and females-photographic documentation; Table S1: Body measurements of *Aeolothrips intermedius* in different crops and localities (μm).
