# Supplementary figures and images for: The Effects of Locality and Host Plant on the Body Size of Aeolothrips intermedius (Thysanoptera: Aeolothripidae) in the Southwest of Poland
Source: Insects. 2019 Aug 22;10(9):266. doi: 10.3390/insects10090266 (PMC6780419; doi:10.3390/insects10090266)

| 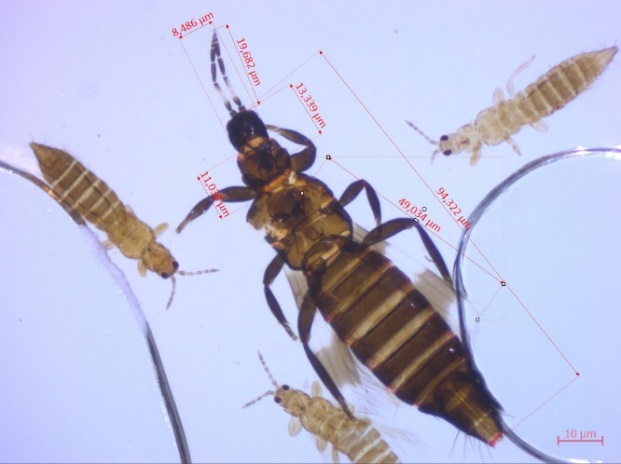A |
| --- |
| 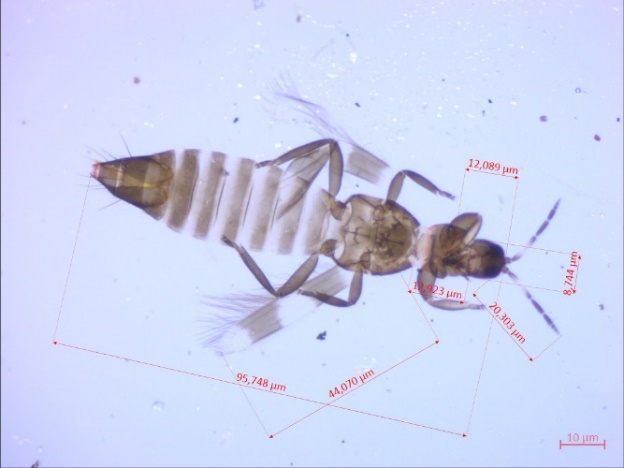B |
| 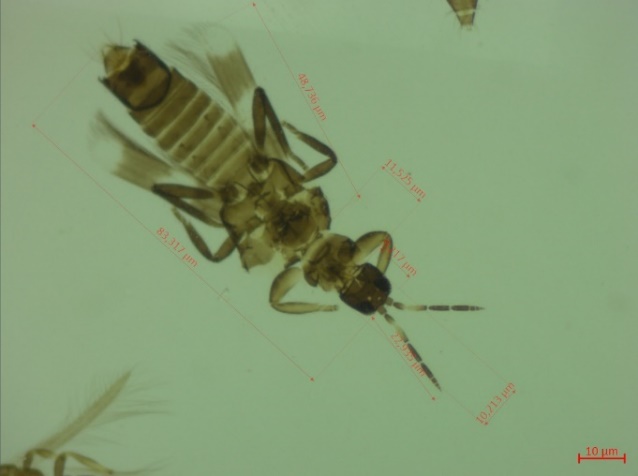C |
| 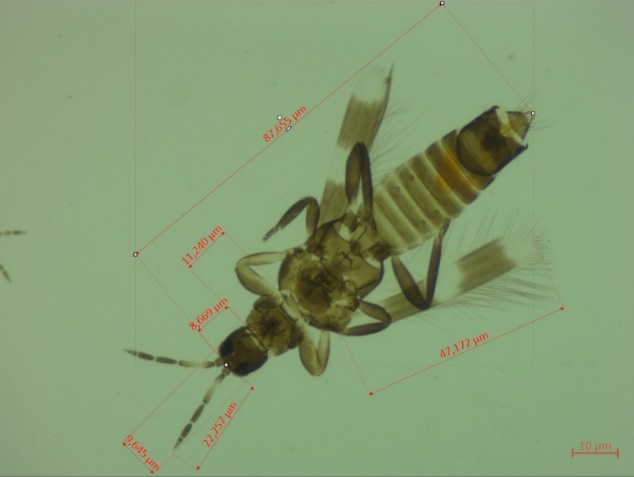D |

Supplement: Supplementary file 1 [file insects-10-00266-s001.zip › Supplementary material 1.docx]
